# Evaluation of hemilaminectomy use in microsurgical resection of intradural extramedullary tumors

**DOI:** 10.3892/ol.2014.1949

**Published:** 2014-03-07

**Authors:** RUI GU, JIA-BEI LIU, PENG XIA, CHEN LI, GUANG-YAO LIU, JIN-CHENG WANG

**Affiliations:** Department of Orthopedics, China-Japan Union Hospital, Jilin University, Changchun, Jilin 130033, P.R. China

**Keywords:** intradural extramedullary tumors, hemilaminectomy, microsurgery, Frankel grade, spinal stability

## Abstract

The aim of this study was to investigate the microsurgical techniques of hemilaminectomy, used in the excision of intradural extramedullary (IDEM) tumors, and to illustrate its clinical effects. Clinical data obtained from 16 patients (seven males, nine females) with IDEM tumors, which were treated at the China-Japan Union Hospital between January 2009 and December 2011, were retrospectively analyzed. The mean age of patients was 49 years, ranging from 34–72 years. The IDEM tumors were located cervically in three patients, thoracically in four patients and at the thoracico-lumbar level in nine patients. Fourteen patients underwent hemilaminectomy, while two patients were treated with laminectomy during surgery. The clinical effect of hemilaminectomy was evaluated based on Frankel grade. The mean bleeding volume was 300 ml (range, 150–500 ml) and the mean duration of surgery was 140 min (range, 90–200 min). The maximum and minimum tumor volumes were 4×1.5×1.5 cm and 1.5×1.0×1.0 cm, respectively. Neurinoma was evident in 11 patients, meningioma in four cases and neurofibroma in one case. Three cases improved from Frankel grade B to C, five cases improved from grade C to D and seven cases improved from grade D to grade E. All patients were followed up for a period of 6–40 months, with a mean follow-up time of 23.7 months. None of the patients exhibited tumor recurrence or spinal instability. The mean bleeding volume of patients that underwent hemilaminectomy and laminectomy was 275 and 475 ml, respectively. The advantages of hemilaminectomy are minor invasion, less bleeding and retention of spinal stability. In general, hemilaminectomy for the excision of IDEM tumors has a satisfactory outcome.

## Introduction

Intradural extramedullary (IDEM) tumors are common types of primary or secondary tumors located in the spinal canal; neurinoma and meningioma are the most common types of IDEM tumor. The most effective treatment of IDEM is achieved through early excision. Laminectomy is the traditional approach used to excise IDEM tumors; however, the stability of the spine is often affected, due to impairment of the posterior column. With the development of microsurgical techniques, to remove the tumor while maintaining the stability of spinal biodynamics with the least amount of invasion is raising more and more concerns. In recent years there have been various studies (1,2) which have investigated the excision of IDEM tumors through hemilaminectomy. Compared with the traditional surgical approach, hemilaminectomy is relatively safer with fewer traumas, and may help maintain the stability of the spine. If surgical indications are interpreted correctly, satisfactory results may be expected (3–5) In the present study, 16 cases of IDEM tumor excision using hemilaminectomy are reported.

## Patients and methods

### General patient information

Sixteen patients (seven males, nine females), with IDEM tumors removed by hemilaminectomy between January 2009 and December 2011, were retrospectively analyzed. The mean patient age was 49 years, ranging from 34–72 years. The patients’ complete medical history ranged from 7 months to 3 years, with a mean of 15.7 months. The IDEM tumors were located at different levels of the spinal canal (at the cervical level in three patients, thoracic level in four and thoracico-lumbar level in nine). According to preoperative Frankel classification, there were three cases classified as grade B, five as grade C, seven as grade D and one as grade E. All patients exhibited symptoms caused by spinal cord compression, which were of varying severity. Twelve patients experienced radicular pain, numbness or zonesthesia; 14 patients suffered from various degrees of limited mobility, below the level of the spinal cord compression; and 15 patients exhibited sensory disturbance of varying severity. All patients were preoperatively examined using magnetic resonance imaging (MRI; Achieva 3.0T TX, Royal Philips Electronics, Amsterdam, The Netherlands) which revealed masses located in the spinal canal. MRI imaging revealed the precise spinal segments in which the tumors were located. This study was conducted in accordance with the Declaration of Helsinki and with approval from the ethics committee of Jilin University (Changchun, China). Written informed consent was obtained from all participants.

### Procedures

**Figure 1:**
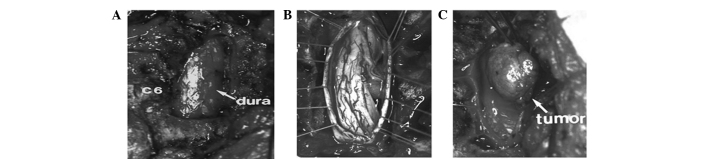
Intraoperative condition of cervical IDEM tumor excised by hemilaminectomy (A) The IDEM was located at C6 level and the dural sac was exposed. (B) Following dural incision, 5-0 sutures were used as traction stitches. (C) The tumor arose laterally from the dura mater. IDEM, intradural extramedullary.

All patients received general anesthetic and were placed in the prone position. Syringe needle markers were inserted into spinal processes under the fluoroscope of the C-arm X-ray machine to assure accurate positioning. A posterior midline longitudinal incision was made and the subcutaneous tissues and lumbodorsal fascia of the affected side were divided. The supraspinal ligaments, interspinal ligaments and tendinous insertions of the contralateral muscles were retained. The paraspinal muscles of the affected side were stripped, exposing the unilateral lamina to the inner edge of the articular process. Corresponding segments of the lamina were removed by a high-speed burr. Ligamentum flavum was removed and the dural sac was exposed. The oblique tilting of the operating table to the contralateral side (typically 15–20 degrees) ensured an adequate surgical field for the procedure. A microscope was positioned to begin microsurgical excision of the IDEM tumors (Fig. 1). An intracapsule excision was made, and then finished the removal of the IDEM tumors between the surrounding normal structures and the tumor. Bipolar coagulation was used to maintain hemostasis, and the dura mater was tightly sutured with 5-0 lines using continuous mattress suture, covered with an absorbable gelatin sponge. Negative pressure drainage was performed in all the cases.. In two cases, tumors were large and located to the rear, thus it was difficult to fully reveal the tumors through hemilaminectomy. Therefore, laminectomy was performed during surgery with internal fixation of the pedicle screw and rod system. In the remaining cases, tumors were successfully removed by hemilaminectomy. Generally, negative-pressure drains were removed within 24–48 h. All 16 patients were confirmed to have IDEM tumors intraoperatively.

## Results

The mean intraoperative bleeding volume was 300 ml (range, 150–500 ml) and the mean surgery time was 140 min (range, 90–200 min). The mean bleeding volume of patients undergoing hemilaminectomy and laminectomy was 275 and 475 ml, respectively. All tumors were resected completely. The maximum tumor volume was 4×1.5×1.5 cm and the minimum was 1.5×1.0×1.0 cm. Neurinoma was identified in 11 patients, meningioma in four cases and neurofibroma in one case. Cerebrospinal fluid leakage occurred in one patient; the fissure was tightly sutured with pressure bandaging in the Trendelenburg position, infection did not occur and the wound healed within 10 days. Patients were followed up for a period of between 6 and 40 months, with a mean follow-up time of 23.7 months. There were no local tumor recurrences or secondary spinal deformities, and spinal stabilities were determined to be satisfactory. All patients achieved remission of symptoms. Pre- and postoperative Frankel grades of the patients are shown in Table I.

## Discussion

Laminectomy is the traditional approach used to excise IDEM tumors. However, as the removal of the posterior lamina, spinous process, supraspinous ligament, interspinous ligament and ligamentum flavum is necessary, the stability of the spine is often affected due to impairment of the posterior column (6–9). Postoperative instability is most likely to occur in the cervical and lumbar segments of the spinal canal (10). Lack of the posterior column and exposure of the dural sac may lead to local fibrosis and scar formation. Seppälä *et al* (11) reported 187 cases of intraspinal neurinoma resection through laminectomy. The rate of total resection was 90%. However, 10% of patients experienced various postoperative complications (including pain, spinal instability and cerebrospinal fluid leakage), and the mortality rate was 1.5%. The incidence of spinal instability following laminectomy has been reported to be 20% in adults (12) and ≤45% (13) in children.

Due to the disadvantages of laminectomy, the use of hemilaminectomy has been proposed for the treatment of IDEM tumors. Chiou *et al* (14) reported that patients undergoing microsurgical resection of intraspinal tumors through hemilaminectomy demonstrated fewer postoperative complications and shorter hospital stays than observed with laminectomy. Yaşargil *et al* (15) recommended the use of hemilaminectomy for the treatment of intraspinal tumors. Oktem *et al* (16) reported 20 cases of resection of intraspinal tumors through hemilaminectomy in 2000, whereby no spinal instability was exhibited during two years of patient follow-up. Yu *et al* (17) reported that excision of tumors by hemilaminectomy resulted in a shorter duration of surgery and less bleeding (P<0.01), compared with that by laminectomy. Naganawa *et al* (18) reported that hemilaminectomy could achieve increased neurofunctional recovery and spinal stability, in 20 patients (mean follow-up period, 85 months) who underwent surgical resection of IDEM tumors through hemilaminectomy. In the present study, no surgical wound infection or spinal instability was observed. Patients could have bed activities (including axial turning and a leg lift) 3 days after surgery and were able to sit up and walk with the assistance of a waist support 5 days after surgery; such clinical effects were similar to those achieved in other reports (8,19).

The spinous process was left in place and therefore the surgical field was relatively small, so surgery was inevitably be affected when adopting hemilaminectomy (18). In order to increase the visual field, damage to facet joints during surgery often occurs. Therefore, it is necessary to preserve the lateral articular processes, as damage to the facet joints is an important factor in spinal instability (20). In the present study, facetectomy was performed in order to fully expose the elongated tumor in one patient that underwent hemilaminectomy with the pedicle screw and rod system.

According to the experience of the authors, when separating the tumor and spinal cord, the tumor should be stripped gently. The spinal cords of some cases may become severely compressed and neither nerve hook nor nerve stripper are permitted to drag the spinal cord during surgery. Complete resectioning of the well-encapsulated tumors is relatively facile. As for tumors that are large, fragile and adhered to the dura mater or located anterolateral to the spinal cord, decompression is performed by piecemeal excision in order to avoid spinal cord damage. For tumors in which the nerve fibers are trapped, it is important to avoid avulsion of the spinal cord which may be caused by forcible traction. The expansive growth of IDEM tumors leads to the dilatation of dura mater, thus, the dura mater may be sutured tightly without tension. When the dural mater is thin or absent, it may be repaired with thoracolumbar fascia or artificial dura mater in order to reduce cerebrospinal fluid leakage. Notably, suturing of the dura mater in a limited operative view requires an adequate amount of practice under a microscope.

In order to obtain complete resection of the tumor, the parent nerve root and tumor could generally be resected together without apparent postoperative dysfunction. Schuhhiess and Gullotta (21) reported that 10 cases of relevant nerve root resection were performed for complete removal of intraspinal neurinomas, whereby no serious or permanent neurodysfunction was observed. However, Celli (22) reported that 26 patients with neurinomas underwent relevant nerve root resection (C5-C8 or L3-S1) whereby four patients exhibited aggravated motor dysfunction and two patients experienced permanent motor dysfunction. In the present study, five patients underwent inseparable parent nerve root resection; two cases in the upper cervical spine and three cases in the thoracic spine. Of those located in the cervical spine, only one patient experienced postoperative ipsilateral shoulder pain, and this gradually improved by itself. Sario-glu *et al* (23) described 40 patients that underwent hemilaminectomy for resection of intraspinal tumors and proposed that hemilaminectomy could be applied to all intraspinal tumor resections, with the exception of bilateral widely invading epidural tumors. In the present study, the majority of the IDEM tumors inclined to one side, thus, satisfactory surgical exposure was achieved. However, two of the IDEM tumors were large, located ventrally and were difficult to fully expose; therefore, laminectomy was performed instead, with pedicle screw and rod systems.

With consideration of the stability of the facet joints, the width of the open surgical window is usually 1.0–1.5 cm, thus, the tumor may not be fully exposed. The authors of the present study argue that the width of the window of the cervical canal should be 2 cm. The window located in the cervical segment is the widest one of the spinal canal, followed by the lumbar segment. Due to the location of the ribs and costotransverse joints, the window width of the thoracic canal is the narrowest. In the current study, since the majority of the IDEM tumors were relatively small and inclined to one side, the width of the surgical window was sufficient. The window width of patients was measured during surgery. Among them, the width of the lower lumbar and upper cervical segments were the widest, while the lower cervical and thoracic segments remained relatively limited. The undercut of the lamina may lead to difficulty in the removal of the tumor and increase the possibility of spinal cord injury. Therefore, hemilaminectomy could not applied to all intraspinal tumor resections. We propose that unilateral IDEM tumors are the most suitable for hemilaminectomy. Additionally, the transverse diameter of the tumor should generally be < 2 cm and the span of the tumor should be limited to a breadth of two vertebra.

In conclusion, microsurgical resection of IDEM tumors by hemilaminectomy may conserve the stability of spinal biodynamics with as little invasion as possible. It is of great importance that the surgical indications are interpreted correctly and complete tumor resectioning is achieved without aggravating the spinal cord injury.

## Figures and Tables

**Table I:** Pre- and postoperative Frankel grades of patients.

	Postoperative Frankel grade				
	
Preoperative Frankel grade	A	B	C	D	E
A	0	0	0	0	0
B	0	0	3	0	0
C	0	0	0	5	0
D	0	0	0	0	7
E	0	0	0	0	1
